# Gender-specific differences in COPD symptoms and their impact for the diagnosis of cardiac comorbidities

**DOI:** 10.1007/s00392-021-01915-x

**Published:** 2021-07-31

**Authors:** Franziska C. Trudzinski, Christina Kellerer, Rudolf A. Jörres, Peter Alter, Johanna I. Lutter, Frederik Trinkmann, Felix J. F. Herth, Marion Frankenberger, Henrik Watz, Claus F. Vogelmeier, Hans-Ulrich Kauczor, Tobias Welte, Jürgen Behr, Robert Bals, Kathrin Kahnert

**Affiliations:** 1 Department of Pneumology and Critical Care Medicine, Thoraxklinik, Translational Lung Research Center Heidelberg (TLRC-H), German Center for Lung Research (DZL), University of Heidelberg, Heidelberg, Germany; 2 School of Medicine, Institute of General Practice and Health Services Research, Technical University of Munich, Munich, Germany; 3 Institute and Outpatient Clinic for Occupational, Social and Environmental Medicine, Comprehensive Pneumology Center Munich (CPC-M), German Center for Lung Research (DZL), Ludwig Maximilians University (LMU), Munich, Germany; 4 Department of Medicine, Pulmonary and Critical Care Medicine, German Center for Lung Research (DZL), Philipps University of Marburg (UMR), Marburg, Germany; 5 Institute of Health Economics and Health Care Management, Comprehensive Pneumology Center Munich (CPC-M), German Center for Lung Research (DZL), Helmholtz Zentrum München GmbH - German Research Center for Environmental Health, Munich, Germany; 6 University Munich, Asklepios Hospital Gauting and Helmholtz Zentrum München, Comprehensive Pneumology Center (CPC-M), German Center for Lung Research (DZL), Ludwig-Maximilians University (LMU), Munich, Germany; 7 Pulmonary Research Institute at LungenClinic Grosshansdorf, Airway Research Center North (ARCN), German Center for Lung Research, Grosshansdorf, Germany; 8 Department of Diagnostic and Interventional Radiology, Translational Lung Research Center Heidelberg (TLRC-H), German Center for Lung Research (DZL), University Hospital of Heidelberg, Heidelberg, Germany; 9 Department of Pneumology, Biomedical Research in Endstage and Obstructive Lung Disease Hannover (BREATH), Member of the German Center for Lung Research (DZL), Hannover Medical School, Carl-Neuberg-Str. 1, 30625 Hannover, Germany; 10 Department of Internal Medicine V, Pulmonology, Allergology, Critical Care Care Medicine, Saarland University Hospital, Homburg, Germany; 11 Department of Internal Medicine V, Comprehensive Pneumology Center (CPC-M), German Center for Lung Research (DZL), University Hospital, Ludwig-Maximilians University (LMU), Munich, Germany

**Keywords:** COPD, Gender, COPD assessment test, Cardiac comorbidities, Symptoms

## Abstract

**Background:**

In chronic obstructive pulmonary disease (COPD), gender-specific differences in the prevalence of symptoms and comorbidity are known.

**Research question:**

We studied whether the relationship between these characteristics depended on gender and carried diagnostic information regarding cardiac comorbidities.

**Study design and methods:**

The analysis was based on 2046 patients (GOLD grades 1–4, 795 women; 38.8%) from the COSYCONET COPD cohort. Assessments comprised the determination of clinical history, comorbidities, lung function, COPD Assessment Test (CAT) and modified Medical Research Council dyspnea scale (mMRC). Using multivariate regression analyses, gender-specific differences in the relationship between symptoms, single CAT items, comorbidities and functional alterations were determined. To reveal the relationship to cardiac disease (myocardial infarction, or heart failure, or coronary artery disease) logistic regression analysis was performed separately in men and women.

**Results:**

Most functional parameters and comorbidities, as well as CAT items 1 (cough), 2 (phlegm) and 5 (activities), differed significantly (*p* < 0.05) between men and women. Beyond this, the relationship between functional parameters and comorbidities versus symptoms showed gender-specific differences, especially for single CAT items. In men, item 8 (energy), mMRC, smoking status, BMI, age and spirometric lung function was related to cardiac disease, while in women primarily age was predictive.

**Interpretation:**

Gender-specific differences in COPD not only comprised differences in symptoms, comorbidities and functional alterations, but also differences in their mutual relationships. This was reflected in different determinants linked to cardiac disease, thereby indicating that simple diagnostic information might be used differently in men and women.

**Clinical trial registration:**

The cohort study is registered on ClinicalTrials.gov with identifier NCT01245933 and on GermanCTR.de with identifier DRKS00000284, date of registration November 23, 2010. Further information can be obtained on the website http://www.asconet.net.

**Supplementary Information:**

The online version contains supplementary material available at 10.1007/s00392-021-01915-x.

## Introduction

Chronic obstructive lung disease (COPD) has a high prevalence worldwide [1] and is known to be associated with multiple comorbidities, in particular cardiovascular disorders [2, 3]. Many studies have shown differences between males and females regarding the prevalence of COPD and comorbidities [4, 5], which might be due to differences in risk factors, or intrinsic, physiological differences [6]. On the other hand, social and behavioural factors also play a role, which includes patients’ reporting of symptoms and disorders as well as tendencies of physicians to consider specific disorders differently in men and women [7].

The diagnosis of comorbidities is of importance for the clinical course and treatment of COPD [1]. Noteworthy, some information on comorbidities and COPD phenotypes can be derived from simple information such as the categorization into GOLD groups, or the modified Medical Research Council (mMRC) scale, or single questions of the COPD Assessment Test (CAT) [8–10]. The fact that symptoms of cardiac disease and COPD show significant overlap, renders it difficult to get clues on cardiac disorders from symptoms alone. Despite this, a comprehensive analysis of cardiac data including echocardiographic measures revealed that residual effects of cardiac disorders on COPD symptoms can be detected, although symptoms are dominated by the lung disease [2]. Whether the role of symptoms and functional parameters for the diagnosis of cardiac disease differs between men and women, is currently not known.

Based on these considerations, we studied whether COPD symptoms assessed by the easily available tools CAT and mMRC showed relationships to common COPD comorbidities and functional alterations that differed between men and women, focussing on functional assessments that are common and do not require special equipment. Regarding cardiac disease, we additionally aimed to reveal whether to the sets of statistical predictors depended on gender. The dataset used was that of the German large multi-center COPD cohort COSYCONET (COPD and Systemic Consequences-Comorbidities Network) [11].

## Methods

### Study population

In total, 2741 patients of age ≥ 40 years with stable COPD were enrolled in COSYCONET; details on the assessments and protocol can be found elsewhere [11]. The present analysis used data from the baseline visit (V1) of patients of spirometric GOLD grades 1–4 [1], who had complete data regarding CAT items and mMRC.

### Pulmonary function tests

According to the COSYCONET study protocol [11], pulmonary function tests (spirometry, body plethysmography, CO diffusing capacity) were performed after inhalation of 400 µg salbutamol and 80 µg ipratropium bromide, and all tests followed established recommendations [11]. Spirometry included the determination of forced expiratory volume in 1 s (FEV_1_), forced vital capacity (FVC) and their ratio FEV_1_/FVC. From body plethysmography, we used RV/TLC, i.e., the ratio of residual volume (RV) to total lung capacity (TLC) that had turned out as particularly informative in previous studies [12]. Moreover, the transfer factor for carbon monoxide (TLCO) from a single-breath manoeuver was used. For RV/TLC, the ratio was directly used, as it is known determinants age and sex were included in the regression analyses. The 6-min walk distance (6MWD) was also determined following established protocols [11].

### Questionnaires

Symptoms were assessed by the modified Medical Research Council (mMRC) scale [10] and the COPD Assessment Test CAT [9], whereby the total score and its eight single items were considered; the questions are listed in see e-Tables 1 and 2 in the online data supplement. Patients were categorized into the four GOLD groups (A: low risk, less symptoms; B: low risk, more symptoms; C: high risk, less symptoms; D: high risk, more symptoms) based on both mMRC and CAT, as well as exacerbation history [1].

### Comorbidities

Comorbidities were assessed in structured interviews, whereby additional information on the presence of comorbidities was obtained by the evaluation of disease-specific medication, wherever possible [11, 13]. Kidney function was quantified using the estimated glomerular filtration rate (eGFR), based on the creatinine equation from the Chronic Kidney Disease Epidemiology Collaboration (CKD-EPI) [14]. The diagnoses of the three cardiac diseases coronary artery disease (CAD), heart failure (HF) and myocardial infarction (MI) relied solely on medical history. They were summarized into the variable “cardiac disease” due to their close relationship and high overlap of medication.

### Statistical analysis

Mean values standard deviations (SD), as well as absolute and relative frequencies, were used to describe the data. The statistical comparison of men and women was performed by *t*-tests, Mann–Whitney-*U*-tests and Chi-squared tests, as appropriate. The relationship between CAT items as dependent variables and their statistical predictors was analyzed by multiple linear regression analyses. TLCO was used as binary category with a cut-off value of 60% predicted, which was close to the median value in the total population; this variable served as a potential indicator of lung emphysema. Moreover, a cut-off value of 60 ml/min was used for eGFR, as common for clinical purposes regarding the presence of renal dysfunction. To identify the role of associations of cardiac disease and symptoms in men and women separately, logistic regression analysis was used, with cardiac disease as outcome. Independent variables were selected on the basis of being widely available in clinical practice, conversely, for example, 6MWD, RV/TLC and TLCO were not used. We thus used the eight single CAT items, mMRC, age, BMI, smoking status, and the *z*-scores of FEV_1_ and FEV_1_/FVC as statistical predictors. The analyses were performed separately for men and women. In these analyses, FEV1 and FEV_1_/FVC were included as *z*-scores [15] to achieve an optimal adjustment for age and sex. In addition to the procedure of inclusion of all variables in the logistic regression analyses, we performed stepwise forward and backward selection, to test for the robustness of the set of significant predictors. Analyses were performed by SPSS Version 26 (IBM Corp., Armonk, NY, USA), assuming a two-sided significance level of 0.05.

## Results

### Basic characteristics

Overall, 2046 participants (795 female) were eligible for analysis (Table 1). Men and women showed significant differences in age, body mass index (BMI), pack years, smoking status, FEV_1_/FVC %predicted, RV/TLC, 6MWD and exacerbation profile, but not in FEV_1_%predicted, TLCO %predicted and GOLD groups (CAT and mMRC) or grades. Men and women also differed in the prevalence of most comorbidities, including asthma and cardiac disease, but not in chronic bronchitis or the binary categories of GFR and TLCO (Table 2). Regarding symptoms, the differences between men and women are shown in Table 3, which indicates that CAT items 1 (cough), 2 (phlegm), 4 (breathlessness), 5 (activities) and 7 (sleeplessness) showed significant gender-specific differences, while the total CAT score and mMRC did not.

**Table 1:** Patients’ characteristics

	All, *N* = 2046	Females, *N* = 795	Males, *N* = 1251	*p*
Age (years)	65.0 ± 8.4	63.8 ± 8.4	65.7 ± 8.3	< 0.001
BMI (kg/m^2^)	26.6 ± 5.2	25.8 ± 5.6	27.1 ± 4.8	< 0.001
Packyears	46.1 ± 36.9	37.8 ± 29.5	51.4 ± 40.1	< 0.001
Smoking status (not active*/active)	1532/514	573/222	959/292	0.020
FEV_1_ (%predicted)	53.2 ± 18.4	53.4 ± 17.9	53.0 ± 18.7	0.654
FEV_1_/FVC	51.6 ± 10.8	52.5 ± 10.4	51.0 ± 11.1	0.003
RV/TLC	0.540 ± 0.108	0.567 ± 0.100	0.523 ± 0.110	< 0.001
TLCO (%predicted)	56.9 ± 21.9	56.0 ± 22.1	57.5 ± 21.7	0.155
6-MWD (m)	419 ± 105	408 ± 104	426 ± 105	< 0.001
Exacerbations (0/1/2/3)	932/110/604/399	316/43/279/157	616/67/325/242	< 0.001
GOLD groups (CAT; A/B/C/D)	231/1093/39/683	75/417/17/286	156/676/22/397	0.066
GOLD groups (mMRC; A/B/C/D)	815/509/265/457	300/192/106/197	515/317/159/260	0.149
GOLD grades (1/2/3/4)	188/887/773/198	74/347/301/73	114/540/472/125	0.945

Mean values and standard deviations, as well as absolute numbers are given. Comparisons between groups were performed by unpaired *t*-tests or Chi-square tests of contingency tables, as appropriate

*Ex or never-smoker

**Table 2:** Comorbidities and surrogate markers

	Females, *N* = 795	Males, *N* = 1251	*p*
Comorbidities			
Asthma*	197/795 (25.3%)	198/1251 (15. 8%)	< 0.001
Chronic bronchitis	490/795 (61.6%)	785/1251 (62.7%)	0.612
Sleep apnea	50/795 (6.3%)	166/1251 (13.2%)	< 0.001
Cardiac disease (at least one)	90/793 (11.3%)	322/1249 (25.8%)	< 0.001
Coronary artery disease	63/795 (7.9%)	256/1251 (20.5%)	< 0.001
Heart failure	30/793 (3.8%)	72/1251 (5.8%)	0.046
Myocardial infarction	26/795 (3.3%)	137/1251 (11.0%)	< 0.001
Hypertension*	407/795 (51.2%)	742/1251 (59.3%)	< 0.001
Osteoporosis*	200/795 (25.2%)	126/1251 (10.1%)	< 0.001
Gastrointestinal disorders*	390/795 (49.1%)	547/1251 (43.7%)	0.018
Mental disorders	223/795 (28.1%)	195/1251 (15.6%)	< 0.001
Surrogate markers			
TLCO < 60%predicted	449/743 (60.4%)	685/1206 (56.8%)	0.114
eGFR < 60 ml/min	73/741 (9.9%)	123/1207 (10.2%)	0.809
LVEF < 50%	47/686 (6.8%)	148/1049 (14.1%)	< 0.001
NT-pro BNP (pg/ml)	264 ± 721	332 ± 515	0.001
Troponin (pg/ml)	5.2 ± 9.3	6.5 ± 9.0	0.002
Cardiovascular medication			
Beta blockers	136/793 (17.1%)	313/1251 (25.0%)	< 0.001
Diuretics	135/793 (17.0%)	277/1251 (22.1%)	0.005
Calcium antagonists	118/793 (14.9%)	213/1251 (17.0%)	0.199
ACE inhibitors	169/793 (21.3%)	391/1251 (31.2%)	< 0.001
AT_1_ receptor antagonists	143/793 (18.0%)	217/1251 (17.3%)	0.691

Absolute numbers and percentages are given. *p* values refer to the comparison between females and males and were derived from Chi-square statistics

*Defined by taking into account disease-specific medication [13]

**Table 3:** Symptom scores

	Female, *N* = 795	Male, *N* = 1251	*p*
CAT 1 (cough)	2.17 ± 1.19	2.36 ± 1.15	0.001
CAT 2 (phlegm)	2.09 ± 1.25	2.36 ± 1.28	< 0.001
CAT 3 (chest tightness)	1.91 ± 1.39	1.80 ± 1.36	0.141
CAT 4 (breathlessness)	3.85 ± 1.15	3.72 ± 1.16	0.007
CAT 5 (activities)	2.55 ± 1.49	2.19 ± 1.48	< 0.001
CAT 6 (confidence)	1.06 ± 1.39	0.93 ± 1.28	0.100
CAT 7 (sleeplessness)	2.08 ± 1.50	1.93 ± 1.49	0.028
CAT 8 (energy)	2.49 ± 1.27	2.45 ± 1.25	0.482
CAT total score	18.20 ± 7.25	17.74 ± 7.37	0.157
mMRC	1.60 ± 0.88	1.57 ± 0.91	0.329

Mean values and standard deviations are shown to illustrate the direction of differences, which were not recognizable from median values and quartiles that were often equal. The comparisons between groups were performed by the Mann–Whitney-*U*-test

### Associations of CAT items with clinical characteristics and comorbidities

The relationship between the eight single CAT items, their summary score and mMRC was analyzed as function of potential influencing variables by regression analysis. We omitted those comorbidities, which were part of, or highly overlapping with other comorbidities, such as reflux relative to gastrointestinal disorders. In the first analyses, we kept gender as additional covariate. The results are shown as heat-map in the supplemental e-Fig. 1. In accordance with Table 3, items 1 (cough), 2 (phlegm) 5 (activities) and 8 (energy), as well as the total CAT score, showed significant differences in their mean level between men and women (*p* < 0.05 each), even when adjusting for a broad set of covariates. In contrast, mMRC was not dependent on gender even after multiple adjustments.

**Fig. 1:**
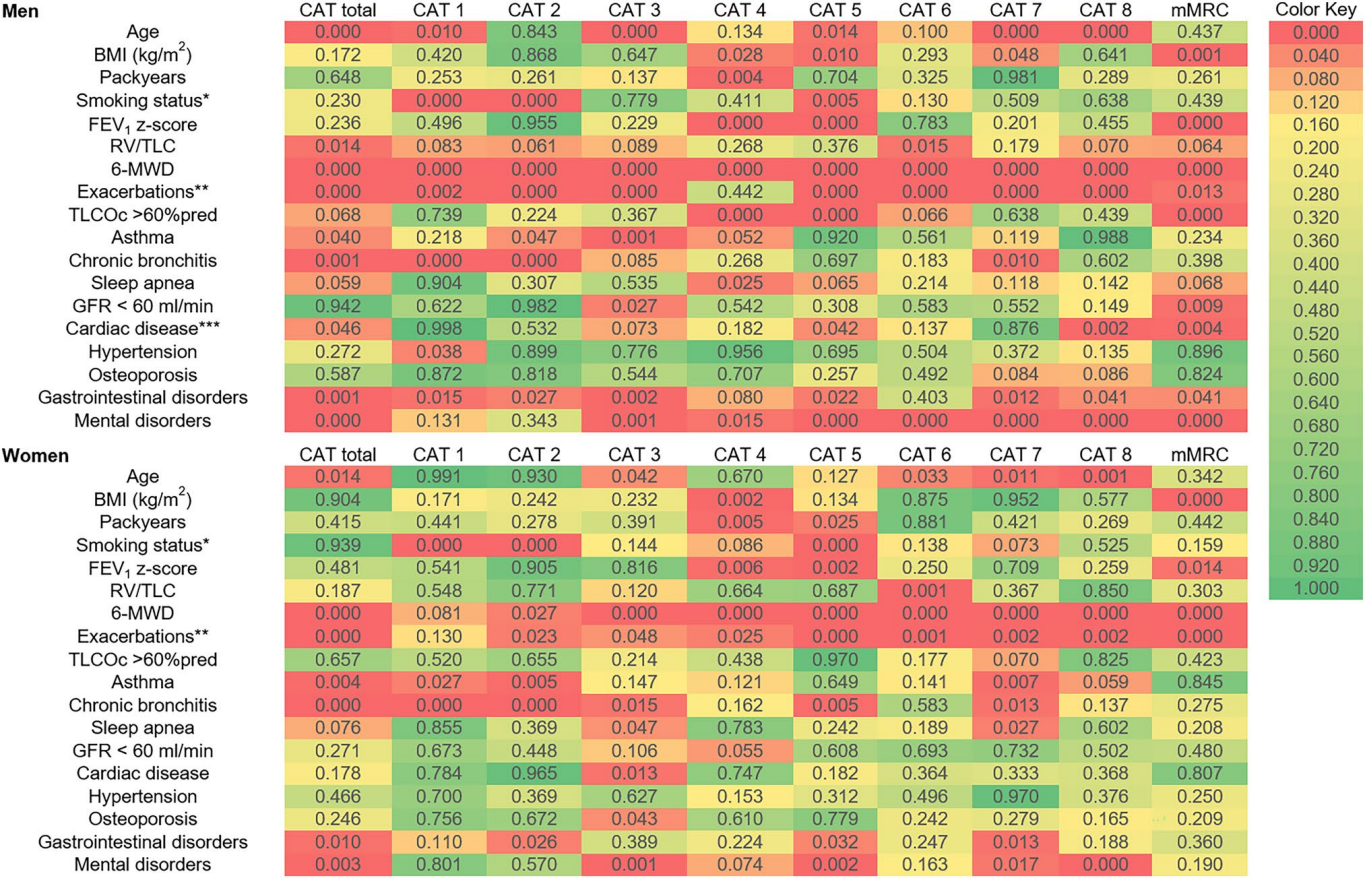
Heat-map of associations of CAT items and mMRC with clinical characteristics and comorbidities for men and women. The figure illustrates the associations between CAT total score, single CAT items and mMRC score to clinical characteristics, lung function, exacerbation history and comorbidities. The heat-map shows *p* values as derived from multiple regression analyses for men and women separately. Colors indicate the strength of the associations (from green. *p* ≥ 0.05. to dark red. strongly significant. *p* ≤ 0.0001). CAT 1 (cough), CAT 2 (phlegm), CAT 3 (chest tightness), CAT 4 (breathlessness), CAT 5 (activities), CAT 6 (confidence), CAT 7 (sleeplessness), CAT 8 (energy). *Current vs. ex or never. **Score values 0–1 vs. 2–3. ***Coronary artery disease, or heart failure, or myocardial infarction. *N* = 2046, males *N* = 1251, females *N* = 795

Analyses were then repeated for men and women separately (Fig. 1). A visual comparison of the two heat-maps indicates that for some of the symptom items the relationships to covariates differed between men and women. We did not perform the comparison via statistical interaction terms, as this greatly blew up the number of parameters and enlarged variance. Gender-specific differences in the associations were obvious for age, BMI, pack years, reduced TLCO, asthma, chronic bronchitis, sleep apnea, cardiovascular disease, gastrointestinal disorders and mental disorders. In some instances, such as hypertension and osteoporosis, associations were weak and comparisons inconclusive.

Considering single CAT items, item 1 (cough) appeared to show different relationships in men and women to age, 6MWD and asthma, item 3 (chest tightness) to asthma and gastrointestinal disorders, item 4 (breathlessness) to reduced TLCO and sleep apnea, item 5 (activities) to age, BMI, reduced TLCO and chronic bronchitis, item 6 (confidence) to age and mental disorder, item 7 (sleeplessness) to sleep apnea and asthma, and item 8 (energy) to cardiac disease and gastrointestinal disorders. Thus, all items except item 2 (phlegm) appeared to show different associations in men and women.

### Cardiac disease related to symptoms and clinical characteristics

In men, age, BMI, smoking status, FEV_1_, FEV_1_/FVC, CAT item 8 and mMRC were significantly (*p* < 0.05 each) associated with cardiovascular disease (Table 4). These findings were robust against stepwise forward or backward selection. In women, only age showed a significant association (Table 4), however in stepwise forward or backward selection also CAT item 5 (activities) became a significant determinant.

**Table 4:** Results of logistic regression analyses regarding cardiac disease as outcome

Covariate	Men						Women					
	Coefficient *B*	SE	*p*	Exp(*B*)	95% CI lower	95% CI upper	Coefficient *B*	SE	*p*	Exp(*B*)	95% CI lower	95% CI upper
Age (years)	0.052	0.010	< **0.001**	1.053	1.032	1.075	0.043	0.017	**0.011**	1.044	1.010	1.079
BMI (kg/m^2^)	0.044	0.015	**0.003**	1.045	1.015	1.075	0.003	0.022	0.874	1.003	0.962	1.047
Smoker (active)	0.397	0.170	**0.020**	1.487	1.065	2.076	0.348	0.281	0.216	1.416	0.816	2.458
CAT 1 (cough)	− 0.085	0.091	0.354	0.919	0.768	1.099	0.071	0.141	0.615	1.073	0.815	1.414
CAT 2 (phlegm)	0.069	0.081	0.398	1.071	0.914	1.255	− 0.044	0.132	0.738	0.957	0.739	1.239
CAT 3 (chest tightness)	0.069	0.063	0.272	1.072	0.947	1.213	0.100	0.103	0.331	1.106	0.903	1.354
CAT 4 (breathlessness)	− 0.043	0.084	0.609	0.958	0.812	1.130	− 0.153	0.149	0.305	0.859	0.641	1.149
CAT 5 (activities)	0.055	0.076	0.473	1.056	0.910	1.226	0.221	0.136	0.104	1.247	0.956	1.627
CAT 6 (confidence)	− 0.015	0.069	0.828	0.985	0.861	1.127	− 0.015	0.103	0.881	0.985	0.805	1.204
CAT 7 (sleeplessness)	− 0.067	0.058	0.245	0.935	0.835	1.047	0.083	0.090	0.356	1.086	0.911	1.295
CAT 8 (energy)	0.215	0.077	**0.005**	1.240	1.066	1.442	0.005	0.125	0.967	1.005	0.786	1.285
mMRC	0.227	0.104	**0.029**	1.255	1.023	1.540	0.074	0.174	0.670	1.077	0.765	1.516
FEV_1_/FVC *z*-score	0.349	0.096	< **0.001**	1.417	1.175	1.709	0.253	0.164	0.123	1.288	0.934	1.777
FEV_1_ *z*-score	− 0.264	0.105	**0.012**	0.768	0.626	0.943	− 0.256	0.160	0.110	0.774	0.566	1.060
Constant	− 6.363	1.024	< 0.001				− 5.619	1.637	0.001			

*95% CI* 95% confidence interval, *SE* standard error of regression coefficient B, *Exp(B)* odds ratio per unit change in covariate

## Discussion

The present study investigated whether men and women with COPD showed a different pattern of relationships between symptoms versus clinical and functional alterations and comorbidities. To quantify symptoms, we used CAT and mMRC as well-established instruments [9, 10]. In addition to known differences in prevalence and severity, we observed differences in associations, whereby single questionnaire items were more informative than the total score. Regarding CAT, the levels of cough, phlegm and activities differed between men and women but also their relationship to anthropometric data, lung function, 6MWD and comorbidities. This also applied to other CAT items. For example, cardiac disease was weakly linked to chest tightness in women, but to activities and energy in men in whom also mMRC was relevant. We then used these findings for the statistical prediction of cardiac disease. Only age was a common determinant in men and women, while there were differences among the relevant symptoms. In men, energy and mMRC, BMI, smoking status and spirometric lung function were relevant predictors, while in women the additional predictors only included activities. These observations underline the different role of clinical and functional measures in men and women for getting diagnostic hints to comorbidities in COPD.

Differences in functional measures, comorbidities and symptoms between men and women with COPD have been described in many studies [4, 5], particularly with regard to cardiac comorbidities, sleep apnea, asthma and osteoporosis, and to COPD symptoms such as cough and phlegm []. We quantified symptoms via the single CAT items, since we had found that these carried more information on COPD characteristics than the total score [8]. Indeed, the inspection of Fig. 1 demonstrates the discordances between men and women regarding the correlation with covariates, as well as between single items and total score. We restricted the analyses to CAT and mMRC, as these instruments are compact and in widespread use, whereas the inclusion of the St. George’s Respiratory Questionnaire (SGRQ) would be cumbersome. When tentatively included into the analysis, it led to very complex results, with no improvement in predictive accuracy. Our approach was directed towards measures simple enough to be used in clinical routine. This was also the reason why we did not include biomarkers such as troponin and NT-pro-BNP, despite being predictive for COPD mortality [16].

Regarding CAT items 1, 2 and 5, the differences in their level and relationship to functional and clinical measures might have several underlying causes. Cough and phlegm (CAT 1 and 2) probably involve social factors such as their perception and the willingness to report them. A further factor might be a different prevalence of comorbidities, especially asthma. Cough was linked to chronic bronchitis in men and women, but to asthma only in women. In contrast, phlegm was linked to chronic bronchitis and asthma in both. Differences in smoking might also have played a role, as smoking status was relevant for cardiac diseases only in men. Another symptom different in men and women, was activity (CAT item 5), which was related to BMI and a reduction in CO diffusing capacity in men but not in women. It is well possible that the question “I am not limited to doing any activities at home” is differently interpreted by men and women, as women traditionally perform more work at home and thus might feel limitations stronger at the same level of functional impairments.

Our focus on different correlation structures between symptoms versus functional status and comorbidities had the aim to reveal gender-specific differences beyond the known differences in prevalence or level. Overall, the pattern of correlations was similar in men and women, which should be not surprising in view of the strong effect of functional limitations on symptoms in COPD. The similarities were reflected in the fact that 6MWD, the severity of exacerbations, smoking status, airway obstruction in terms of FEV_1_, air trapping in terms of RV/TLC and osteoporosis showed similar associations. On the other hand, the heat-maps shown in Fig. 1 illustrate marked differences in the relationship of CAT items to covariates for age, BMI, pack years, diffusing capacity, asthma, chronic bronchitis, sleep apnea, cardiac disease, hypertension and gastrointestinal disorders.

The average COPD patient is as likely to die from a cardiovascular cause as from a respiratory cause [17]. With cardiac diseases having a major impact on prognosis [16, 18], we focused the further analysis on these diseases, combining them into a combined variable mainly for reasons of statistical power. As expected, men showed a higher prevalence of cardiac diseases, but the most obvious difference was that CAT item 8 (energy) correlated with cardiac disease in men but not in women. Accordingly, item 8 appeared as significant in the logistic regression analyses (Table 4) in men only. The result is of interest as women are known to announce cardiac diseases with less indicative symptoms. In women, CAT item 5 (activities) was predictive according to logistic regression analysis, in addition to age (Table 4).

Previous data from COSYCONET revealed underdiagnosis and undertreatment of cardiovascular diseases in COPD patients [2], possibly due to shared symptoms of respiratory and cardiac disease. COPD patients may even receive less guideline-based treatment for cardiac diseases [19–21] compared to patients without COPD. Although current treatment recommendations for COPD explicitly refer to concomitant diseases, they do not give a clear recommendation as to when and how the screening for cardiac comorbidities should be performed. Various working groups have pointed out this weakness and proposed regular assessments including a wide range of diagnostic measures, e.g., ECG, laboratory markers, echocardiography, CT scan, coronary angiography, exercise tolerance or stress tests [22, 23].

At least some of the assessments are, however, time-consuming and cost-intensive, rendering their implementation difficult especially in primary care settings. Our study identified simple, easily ascertainable hints on the presence of cardiac comorbidity in COPD. In men, the statement “I have little energy” were important, in addition to other determinants, in women “my chest feels very tight”, age being a common risk factor. If in an individual patient these symptoms appear discordant to the severity of the respiratory disorder, they give a hint that specific cardiologic diagnostic procedures are justified. These findings underline that for a specific diagnostic benefit from the CAT questionnaire single items are sufficient, whereby it is useful, to acknowledge gender-specific differences.

## Limitations

The present study is a cross-sectional approach and can only report correlations but not causal relationships. On the other hand, the patterns regarding gender-specific differences appeared plausible and more than mere correlations. The low prevalence of cardiac disease in women did not allow the evaluation of potential determinants in the same detail as in men. A similar argument applied for the three single cardiac diseases that we summarized into one category, but this might be secondary given the modest aim of our study to find hints on any cardiac disorder in the COPD patients. Moreover, we omitted extensive questionnaires, such as the SGRQ, to avoid tools that are difficult to transfer into clinical practice. The presence of cardiac disease was derived from patient-reports of diagnoses established by physicians and it was not necessarily guideline-based. As respiratory medication aims to attenuate symptoms and improve the functional state, symptoms would have been greater in the absence of medication but this would have been an unrealistic situation. Regarding our aim to get hints on cardiac disease in COPD, respiratory medication might even have improved the situation, since a cardiac contribution to symptoms became relatively stronger.

## Conclusion

Using data from a large COPD cohort, we observed that COPD symptoms measured by single CAT items and mMRC showed relationships to functional and clinical status as well as comorbidities that differed between men and women. These differences were also apparent in different sets of measures, including symptoms, indicative for cardiac disease in men and women. As a potential application, the findings suggest that in men with COPD, elevated scores of one CAT item (energy) should motivate a cardiovascular diagnostic work-up, while in women the situation is more difficult, as a result of the overall lower prevalence of cardiac diseases.

## Supplementary Information

Below is the link to the electronic supplementary material.

Supplementary file1 (DOCX 72 kb)

Supplementary file2 (DOCX 40 kb)

Supplementary file3 (TIF 437 kb)

## Data Availability

The full dataset supporting the conclusions of this article is available upon request and application from the Competence Network Asthma and COPD (ASCONET, http://www.asconet.net/html/cosyconet/projects).

## Abbreviations

6MWD: 6-Minute walk distance
BMI: Body mass index
CAD: Coronary artery disease
CAT: COPD assessment test
CKD-EPI: Chronic Kidney Disease Epidemiology Collaboration
COPD: Chronic obstructive pulmonary disease
COSYCONET: COPD and Systemic Consequences-Comorbidities Network
eGFR: Estimated glomerular filtration rate
FEV_1_: Forced expiratory volume in one second
FVC: Forced vital capacity
HF: Heart failure
MI: Myocardial infarction
mMRC: Modified Medical Research Council dyspnea scale
RV: Residual volume
SD: Standard deviations
SGRQ: St. George’s Respiratory Questionnaire
TLCO: Transfer factor for carbon monoxide

## References

[1] Vogelmeier CF, Criner GJ, Martinez FJ et al (2017) Global strategy for the diagnosis, management, and prevention of chronic obstructive lung disease 2017 report: GOLD executive summary. Eur Respir J 49(3). 10.1164/rccm.201701 10.1183/13993003.00214-2017 28182564

[2] Alter P, Mayerhofer BA, Kahnert K (2019). Prevalence of cardiac comorbidities, and their underdetection and contribution to exertional symptoms in COPD: results from the COSYCONET cohort. Int J Chronic Obstr Pulm Dis.

[3] Divo M, Cote C, de Torres JP (2012). Comorbidities and risk of mortality in patients with chronic obstructive pulmonary disease. Am J Respir Crit Care Med.

[4] Almagro P, Lopez Garcia F, Cabrera FJ (2010). Comorbidity and gender-related differences in patients hospitalized for COPD. The ECCO study. Respir Med.

[5] Celli B, Vestbo J, Jenkins CR (2011). Sex differences in mortality and clinical expressions of patients with chronic obstructive pulmonary disease. The TORCH experience. Am J Respir Crit Care Med.

[6] Silverman EK, Weiss ST, Drazen JM (2000). Gender-related differences in severe, early-onset chronic obstructive pulmonary disease. Am J Respir Crit Care Med.

[7] de Torres JP, Casanova C, Hernandez C, Abreu J, Aguirre-Jaime A, Celli BR (2005). Gender and COPD in patients attending a pulmonary clinic. Chest.

[8] Marietta von Siemens S, Alter P, Lutter JI (2019). CAT score single item analysis in patients with COPD: results from COSYCONET. Respir Med.

[9] Jones PW, Harding G, Berry P, Wiklund I, Chen WH, Kline LN (2009). Development and first validation of the COPD assessment test. Eur Respir J.

[10] Mahler DA, Wells CK (1988). Evaluation of clinical methods for rating dyspnea. Chest.

[11] Karch A, Vogelmeier C, Welte T (2016). The German COPD cohort COSYCONET: aims, methods and descriptive analysis of the study population at baseline. Respir Med.

[12] Alter P, Orszag J, Kellerer C et al (2020) Prediction of air trapping or pulmonary hyperinflation by forced spirometry in COPD patients: results from COSYCONET. ERJ Open Res 6(3). 10.1183/23120541.00092-2020 10.1183/23120541.00092-2020 PMC7383055 32743009

[13] Lucke T, Herrera R, Wacker M (2016). Systematic analysis of self-reported comorbidities in large cohort studies - a novel stepwise approach by evaluation of medication. PLoS ONE.

[14] Levey AS, Stevens LA, Schmid CH (2009). A new equation to estimate glomerular filtration rate. Ann Intern Med.

[15] Quanjer PH, Stanojevic S, Cole TJ (2012). Multi-ethnic reference values for spirometry for the 3–95-yr age range: the global lung function 2012 equations. Eur Respir J.

[16] Waschki B, Alter P, Zeller T et al (2020) High-sensitivity troponin I and all-cause mortality in patients with stable COPD: an analysis of the COSYCONET study. Eur Respir J 55(2). 10.1183/13993003.01314-2019 10.1183/13993003.01314-2019 31831579

[17] Berry CE, Wise RA (2010). Mortality in COPD: causes, risk factors, and prevention. COPD.

[18] Cuthbert JJ, Kearsley JW, Kazmi S (2019). The impact of heart failure and chronic obstructive pulmonary disease on mortality in patients presenting with breathlessness. Clin Res Cardiol.

[19] Knuuti J, Wijns W, Saraste A (2020). 2019 ESC Guidelines for the diagnosis and management of chronic coronary syndromes. Eur Heart J.

[20] Ponikowski P, Voors AA, Anker SD (2016). 2016 ESC Guidelines for the diagnosis and treatment of acute and chronic heart failure: the Task Force for the diagnosis and treatment of acute and chronic heart failure of the European Society of Cardiology (ESC) Developed with the special contribution of the Heart Failure Association (HFA) of the ESC. Eur Heart J.

[21] Piepoli MF, Hoes AW, Agewall S (2016). 2016 European Guidelines on cardiovascular disease prevention in clinical practice: the Sixth Joint Task Force of the European Society of Cardiology and Other Societies on Cardiovascular Disease Prevention in Clinical Practice (constituted by representatives of 10 societies and by invited experts) Developed with the special contribution of the European Association for Cardiovascular Prevention & Rehabilitation (EACPR). Eur Heart J.

[22] Roversi S, Fabbri LM, Sin DD, Hawkins NM, Agusti A (2016). Chronic obstructive pulmonary disease and cardiac diseases. An urgent need for integrated care. Am J Respir Crit Care Med.

[23] Vanfleteren L, Spruit MA, Wouters EFM, Franssen FME (2016). Management of chronic obstructive pulmonary disease beyond the lungs. Lancet Respir Med.

