# Supplementary material for: Gender-specific differences in COPD symptoms and their impact for the diagnosis of cardiac comorbidities
Source: Clin Res Cardiol. 2021 Jul 31;112(2):177–86. doi: 10.1007/s00392-021-01915-x (PMC9898364; doi:10.1007/s00392-021-01915-x)
Supplement: Supplementary file 1 — Supplementary file1 (DOCX 72 kb) [file 392_2021_1915_MOESM1_ESM.docx]

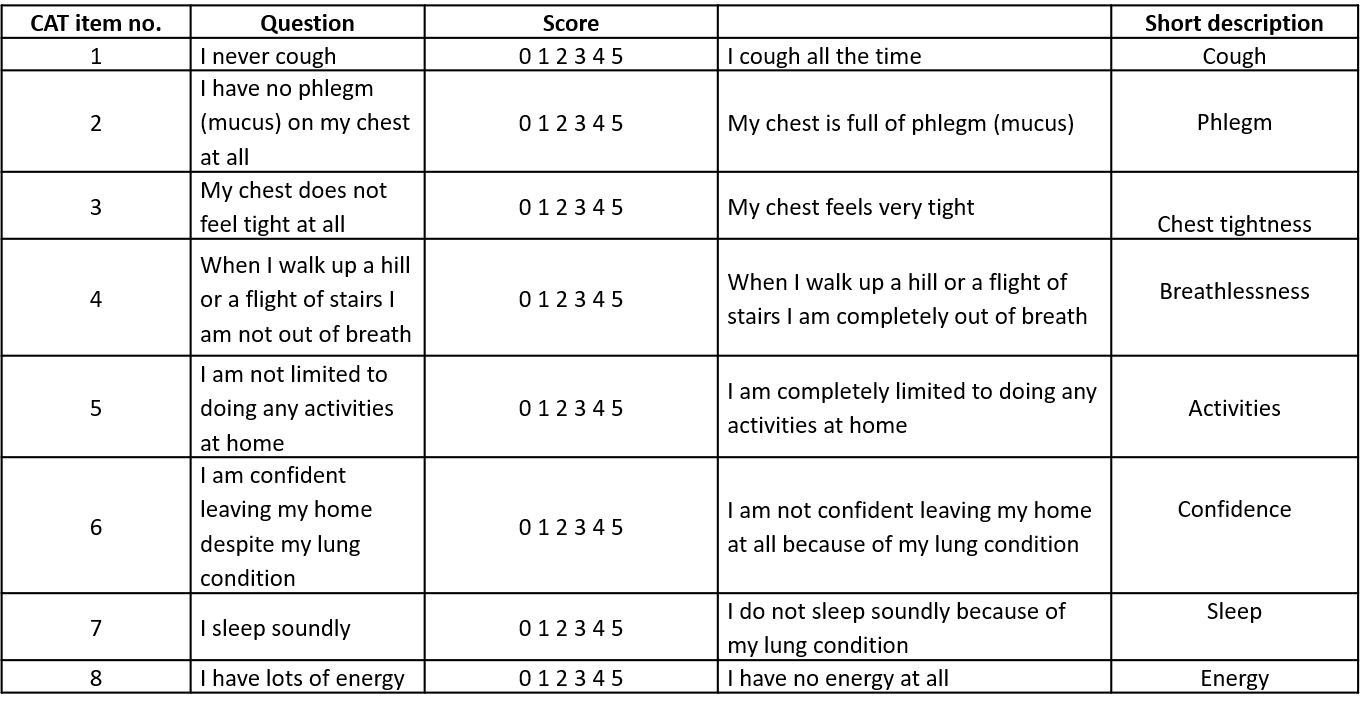


Jones PW, Harding G, Berry P, Wiklund I, Chen WH, Kline Leidy N. Development and first validation of the COPD Assessment Test.

Eur Respir J. 2009;34(3):648-54.

**e-Table 1: COPD Assessment Test (CAT)**
