# Supplementary material for: Gender-specific differences in COPD symptoms and their impact for the diagnosis of cardiac comorbidities
Source: Clin Res Cardiol. 2021 Jul 31;112(2):177–86. doi: 10.1007/s00392-021-01915-x (PMC9898364; doi:10.1007/s00392-021-01915-x)
Supplement: Supplementary file 2 — Supplementary file2 (DOCX 40 kb) [file 392_2021_1915_MOESM2_ESM.docx]

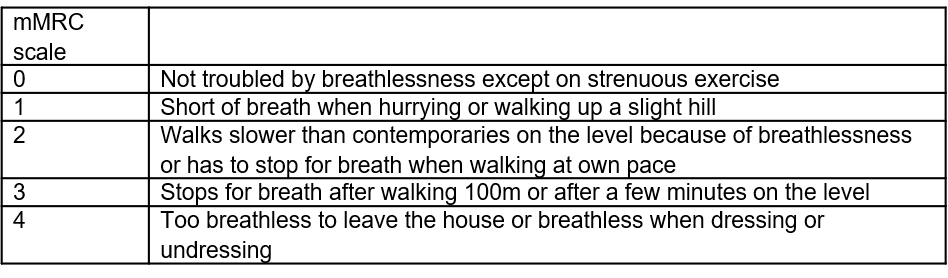


Mahler DA, Wells CK. Evaluation of clinical methods for rating dyspnea. Chest. 1988;93(3):580-6.

This self-rating questionnaire is used to measure the degree of disability that breathlessness poses

on day-to-day activities on a scale from 0 to 4.

**e-Table 2: Modified Medical Research Council (mMRC) scale**
