# Supplementary figures and images for: Gender-specific differences in COPD symptoms and their impact for the diagnosis of cardiac comorbidities
Source: Clin Res Cardiol. 2021 Jul 31;112(2):177–86. doi: 10.1007/s00392-021-01915-x (PMC9898364; doi:10.1007/s00392-021-01915-x)

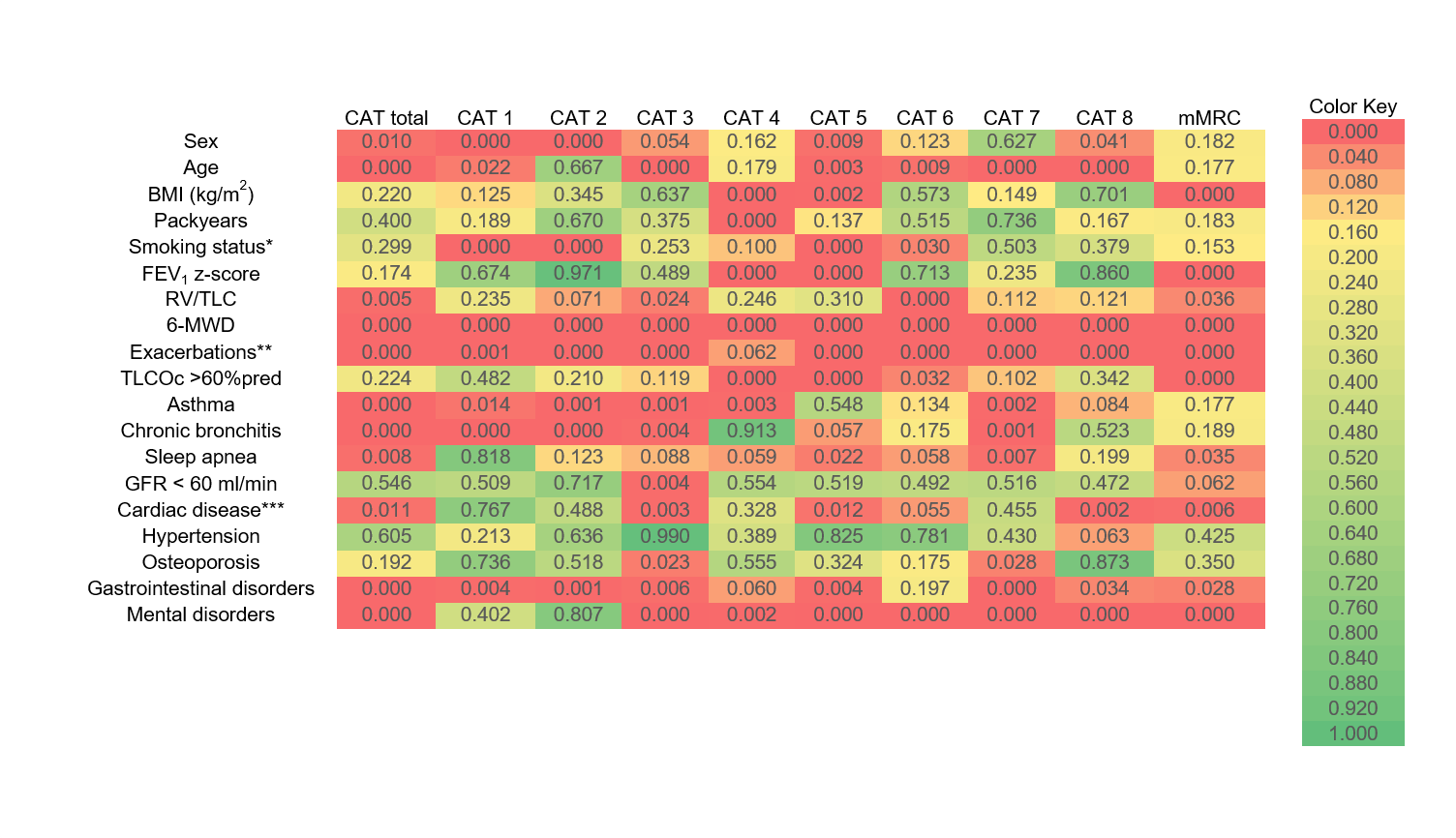

Supplement: Supplementary file 3 — Supplementary file3 (TIF 437 kb) [file 392_2021_1915_MOESM3_ESM.tif]
